# Identification of Novel Genes Associated with Renal Tertiary Lymphoid Organ Formation in Aging Mice

**DOI:** 10.1371/journal.pone.0091850

**Published:** 2014-03-17

**Authors:** Yuan Huang, Christina R. Caputo, Gerda A. Noordmans, Saleh Yazdani, Luiz Henrique Monteiro, Jaap van den Born, Harry van Goor, Peter Heeringa, Ron Korstanje, Jan-Luuk Hillebrands

**Affiliations:** 1 Department of Pathology & Medical Biology - Pathology, University of Groningen, University Medical Center Groningen, Groningen, The Netherlands; 2 Department of Pathology, School of Basic Medical Sciences, Fudan University, Shanghai, China; 3 The Jackson Laboratory, Bar Harbor, Maine, United States of America; 4 Department of Internal Medicine - Nephrology, University of Groningen, University Medical Center Groningen, Groningen, The Netherlands; 5 Department of Pathology & Medical Biology - Medical Biology, University of Groningen, University Medical Center Groningen, Groningen, The Netherlands; UCL Institute of Child Health, United Kingdom

## Abstract

A hallmark of aging-related organ deterioration is a dysregulated immune response characterized by pathologic leukocyte infiltration of affected tissues. Mechanisms and genes involved are as yet unknown. To identify genes associated with aging-related renal infiltration, we analyzed kidneys from aged mice (≥20 strains) for infiltrating leukocytes followed by Haplotype Association Mapping (HAM) analysis. Immunohistochemistry revealed CD45^+^ cell clusters (predominantly T and B cells) in perivascular areas coinciding with PNAd^+^ high endothelial venules and podoplanin^+^ lymph vessels indicative of tertiary lymphoid organs. Cumulative cluster size increased with age (analyzed at 6, 12 and 20 months). Based on the presence or absence of clusters in male and female mice at 20 months, HAM analysis revealed significant associations with loci on Chr1, Chr2, Chr8 and Chr14 in male mice, and with loci on Chr4, Chr7, Chr13 and Chr14 in female mice. *Wisp2* (Chr2) showed the strongest association (P = 5.00×10^−137^) in male mice; *Ctnnbip1* (P = 6.42×10^−267^) and *Tnfrsf8* (P = 5.42×10^−245^) (both on Chr4) showed the strongest association in female mice. Both *Wisp2* and *Ctnnbip1* are part of the Wnt-signaling pathway and the encoded proteins were expressed within the tertiary lymphoid organs. In conclusion, this study revealed differential lymphocytic infiltration and tertiary lymphoid organ formation in aged mouse kidneys across different inbred mouse strains. HAM analysis identified candidate genes involved in the Wnt-signaling pathway that may be causally linked to tertiary lymphoid organ formation.

## Introduction

As healthy individuals age most of them display a gradual decline in renal function as identified by diminished glomerular filtration rate [1]. Various factors influence the rate of decline including the presence of co-morbidities (like hypertension and diabetes mellitus), ethnicity and sex [2]. However, it was previously shown that in a group of healthy subjects one third had no absolute decrease in renal function [3] suggesting that genetic predisposition for decline in renal function, or protection thereof, exists. Aging-related decline in renal function is characterized histopathologically by vascular, glomerular and tubulo-interstitial scarring [4]. The process of progressive renal scarring with age is believed to result from repetitive clinical or silent insults of acute kidney injury, which is accompanied by local and systemic inflammatory processes. The inflammatory cascade initially facilitates regeneration and repair but may promote fibrosis in the chronic phase [5]. Consequently, attenuation of (aging-related) renal inflammation is expected to slow down the process of renal scarring and thereby functional decline.

Microarray analysis of human normal kidney samples revealed increased expression of immune genes at old age among which B and T cell-specific genes including immunoglobulin μ, λ, κ chains and TCRβ, respectively [6]. These data suggest the presence of increased numbers of infiltrating lymphocytes in the aged kidney, which indicate that there is a conserved increase of immune surveillance or inflammation in the kidney with age. Similarly, also kidneys from aged (17–19 months) C57BL6 mice were characterized by increased expression of immune-related genes when compared with young (8–10 weeks) mice [7]. Whether strain-dependent differences exist with respect to aging-related renal inflammation is as yet unknown.

Although it is well recognized that renal aging in both humans [6], [8] and mice [7] is accompanied by an elevated inflammatory status, the cellular and molecular mechanisms underlying this phenomenon are still unknown. Depending on the spatial organization of infiltrating leukocytes in target tissues, the function and consequences may vary. Whereas scattered low level inflammation is considered relatively benign, chronic inflammation can result in tertiary lymphoid organ (TLO) formation which can be associated with tissue damage [9]. As an example, reduced expression of the calcineurin α isoform in mice resulted in massive spontaneous TLO formation in aged mice which inversely correlated with renal function. Attenuation of TLO formation improved kidney function, indicating that the process of TLO formation contributed to the observed nephrotoxicity [10].

As yet, it is unknown whether aging-related renal inflammation and TLO formation is genetically driven. Therefore, we here analyzed aging-related renal inflammation and TLO formation in kidneys collected from healthy aged mice (≥20 inbred strains) followed by Haplotype Association Mapping (HAM) genetic analysis in order to identify associated genes. HAM analysis, also known as *in silico* QTL mapping and similar to genome-wide association studies (GWAS) in humans, is a powerful tool to identify genetic loci and to find associations between phenotype and haplotype in mouse inbred strains [11]. This approach utilizes high-density single-nucleotide polymorphism (SNP) data from many inbred strains to identify chromosomal haplotypes associated with phenotypic traits of interest. The strength of this approach was shown in previous studies in aged mice resulting in the identification of a novel gene involved in the regulation of plasma sodium levels [12] and loci for age-related albuminuria [13]. The loci identified in the latter study were concordant with loci associated with human diabetic nephropathy as identified by GWAS, indicating involvement of common mechanisms in albuminuria development in mice and humans [13].

Our data revealed differential lymphocytic infiltration and TLO formation in kidneys from aged mice across different inbred mouse strains. Subsequent HAM analysis identified candidate genes, which may be causally linked to aging-associated TLO formation.

## Materials and Methods

### Ethics Statement

All experiments were approved by The Jackson Laboratory's Animal Care and Use Committee.

### Mice

Males (20 strains) and females (23 strains) from different mouse inbred strains were obtained from The Jackson Laboratory, Bar Harbor, ME. If any of the mice died during follow-up (up to 20 months), they were replaced with mice from the same strain. Mice were housed in a climate-controlled pathogen-free facility with a 12:12-h light–dark cycle and provided free access to food and water throughout the experiment. After weaning, they were maintained on a chow diet (Lab diet 5K52, PMI Nutritional International, Bentwood, MO, USA). At 20 months, kidneys from 6.5 (median) [3 (min)–13 (max)] male and 6 [3]–[12] female mice per strain were analyzed for the presence of perivascular infiltrates (described below). At 6 and 12 months, kidneys from respectively 5–6 and 4–5 mice were analyzed in a subset of strains.

### Periodic acid-Schiff (PAS) staining and quantitative analyses

For morphological analysis, kidney and liver tissue was fixed in Bouin's fixative followed by embedding in paraffin. Paraffin-embedded tissue blocks were cut into 2 μm sections, and Periodic acid-Schiff (PAS) staining was performed for histological analysis. Because the size of the immune cell clusters depended on the way the tissue was cut and their localization, we measured them as follows in renal tissue: 1) Perivascular clusters which were found around the blood vessels at the renal hilum area were excluded; 2) The total number of perivascular clusters per kidney section as well as the total cumulative size of the clusters were determined. The latter was done using the following equation: relative cluster size  =  (total cumulative cluster area/total renal tissue area) ×100%; 3) male and female mice were measured in the same way, while analyzed separately. For quantification of the number of renal lymph vessels the cortical area was selected in each kidney and the number of all podoplanin^+^ (see below) vessel-like structures with clear lumen were counted and expressed as the number of lymph vessels per mm^2^ of cortical area.

### Immunohistochemistry

For immunohistochemical staining, sections were deparaffinized in xylene followed by rehydration. Heat-induced antigen retrieval was performed in a microwave in 10 mM sodium citrate buffer (pH 6.0) followed by an endogenous avidin and biotin blocking step (Avidin/Biotin Blocking Kit, Vector Laboratories). Kidney sections were stained for rat anti-mouse CD45 (pan leukocyte marker, clone 30-F11, BD Biosciences), rabbit anti-human CD3 (T cells, DAKO), rat anti-mouse B220 (B cells, tissue culture supernatant from clone RA3-3A1), rabbit anti-human Ki67 (proliferating cells, NCL-Ki67p, Novocastra - Leica Microsystems B.V.), anti-mouse peripheral node addressin [PNAd] (HEVs, clone MECA-79, Biolegend), hamster anti-mouse podoplanin (clone 811, Acris Antibodies Inc), WISP2 (ABIN709676, Antibodies-Online), TNFRSF8 (ABIN1385704, Antibodies-Online), and CTNNBIP1 (ABIN753748, Antibodies-Online). Liver sections were stained for CD3 and B220 only. Sections were incubated with primary antibodies for either 1 h (podoplanin, WISP2, TNFRSF8, CTNNBIP1) or 2 h at room temperature (CD3, B220, PNAd, Ki67), or overnight at 4°C (CD45). Sections incubated with primary antibodies against Ki67, CD3, B220 and CD45 were then exposed to appropriate biotin-labeled secondary antibodies: anti-rat IgG, anti-rabbit IgG, anti-mouse IgG (DAKO) and anti-rat IgM (Abcam), followed by incubation with peroxidase-conjugated streptavidin for 30 min at room temperature. For detection of podoplanin, sections were incubated with peroxidase-conjugated goat anti-Syrian hamster (Abcam) secondary antibody for 30 min. For detection of WISP2, TNFRSF8, CTNNBIP1, sections were incubated with peroxidase-conjugated goat anti-rabbit secondary antibody (DAKO) and rabbit anti-goat tertiary antibody (DAKO), each for 30 min. Immunoreactivity was visualized using 3,3′-diaminobenzidine (DAB) solution (1 mM DAB, 50 mM Tris–HCl buffer (pH 7.6), 10 mM sodium azide, 0.006% H_2_O_2_) or by adding the substrate 3-amino-9-ethylcarbazole (AEC) from DAKO Envision kit (DAKO). Hematoxylin was used as nuclear counterstaining except the podoplanin-stained sections on which PAS-counterstaining was performed. Negative controls for immunostaining were performed by adding the same concentration of appropriate isotype control antibodies (DAKO) instead of primary antibodies. No specific immunoreactivity was detected in these negative control sections (not shown). Images were captured using a Hamamatsu NanoZoomer 2.0-HT Virtual Slide Scanner (Hamamatsu Photonics, Japan). Quantitative analyses of cluster size were performed using Aperio ImageScope version 10.2.2.2352 image analysis software.

### Immunofluorescence

Four-micrometer thick frozen sections (*C57BL6/J* mouse kidney) were fixed in acetone (10 min., room temperature) and subsequently incubated in 0.03% H_2_O_2_ (in PBS). Sections were pretreated with normal mouse serum (20 min., room temperature), and next incubated for 1 hr with primary antibody mixture consisting of hamster anti-mouse podoplanin (clone 811) with either goat anti-mouse VEGFR3 (R&D Systems) or rabbit anti-mouse LYVE-1 (kind gift from Prof. David Jackson, John Radcliffe University Hospital, Oxford, UK) diluted in PBS/1% BSA. Binding of primary antibodies was detected by incubating the sections for 30 min. with secondary polyclonal antibodies diluted in PBS +1% normal mouse serum: peroxidase-conjugated goat anti-Syrian hamster (Abcam) with FITC-conjugated rabbit anti-goat (DAKO) or FITC-conjugated goat anti-rabbit (DAKO). Peroxidase-activity was visualized using the TSA Tetramethylrhodamine System (PerkinElmer LAS Inc., USA). Sections were mounted in Vectashield mounting medium and analyzed on a Leica DM4000B microscope (Leica Microsystems B.V.).

### Haplotype Association Mapping (HAM) analysis

To identify loci associated with the presence of perivascular immune cell clusters HAM analysis was performed using the Efficient Mixed Model Association (EMMA; http://mouse.cs.ucla.edu/emma) method to control for genetic relevance [14]. In both sexes, binary data (*i.e*. presence or absence) were used based on the threshold at 0.15 of relative cluster size. Strains with relative cluster size <0.15 were marked as “0”, and those >0.15 were marked as “1”. Associations with a P-value <10^−6^ were considered significant. HAM results are displayed in both Manhattan plots (to illustrate the observed associations along the genomic coordinates), and Quantile-Quantile (Q-Q) plots (illustrate deviation of the observed from the expected probability distribution).

### Single Nucleotide Polymorphism (SNP) genotyping

To determine the genotypes of the *Wisp2* and *Tnfrsf8* SNPs for the strains included in our study for which no data were available in the Sanger database (www.sanger.ac.uk/resources/mouse/genomes/) oligonucleotide primers were designed that enabled us to amplify *Wisp2* exon 4 and *Tnfrsf8* exon 5. High quality DNA for all the strains was purchased from The Jackson Laboratory's DNA resource (www.jax.org/dnares/). PCR and subsequent sequencing of the PCR products were performed using standard protocols.

### Statistical analysis

Statistical analysis including calculation of mean distribution and standard error for the cluster size study was carried out using GraphPad Prism 5 software (GraphPad Software Inc., La Jolla, CA, USA). To compare multiple conditions, statistical significance was calculated by one-way ANOVA. The Student *t-test* was used to compare two conditions using the original data. Pearson correlation was used to analyze the mean relative cluster size and number between female and male mice. Pearson's χ^2^ test was performed to analyze the association between kidney and liver perivascular infiltration. A value of P<0.05 was considered to indicate significance.

## Results

### Presence and size of immune cell clusters in aged mice

**Figure 1:**
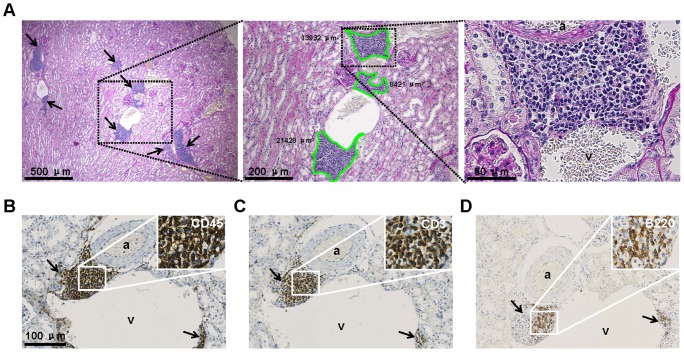
Phenotypic characterization of perivascular immune cell clusters in the aged mouse kidney. (A) PAS staining was used for computerized morphometric analysis. Left panel: low-power magnification (40x) showing representative perivascular infiltrates (arrows) in a male C57L/J mouse. Middle panel: higher-power magnification (100x) of the left panel showing three individually measured perivascular cell clusters. The cell clusters were encircled and the surface area was calculated and expressed in μm^2^ as indicated. Right panel: high-power magnification (400×) of the middle panel. (B) The immune cell clusters consisted of CD45^+^ cells of which the majority was CD3^+^ T cells (C) and B220^+^ B cells (D). Panels B, C & D display serial sections (magnification: 200×). Insets show high-power magnifications (500x) of the indicated areas. Arrows indicate immune cell clusters. a: arteriole, v: vein.

In the vast majority of aged (20 months) kidneys from the 23 strains of female and 20 strains of male inbred mice, infiltrated inflammatory cells were found (Table 1). These inflammatory cells were primarily aggregated in clusters around the veins and arterioles (Figure 1A) whereas the glomeruli and tubulo-interstitium only contained sparse infiltrated cells. To determine the composition of these cell clusters, sections were stained with the pan-leukocyte marker CD45, which is present on all hematopoietic cells, except erythrocytes and plasma cells. CD45 staining showed abundant expression in the renal perivascular cell clusters, indicating that these clusters were dominantly composed of leukocytes (Figure 1B). Staining for CD3 (Figure 1C) and B220 (Figure 1D) revealed that the CD45^+^ cell clusters consisted primarily of T cells and to a lesser extent B cells.

**Table 1:** Mean perivascular cell cluster number and cumulative cluster size identified in male and female mice of the various mouse strains at the age of 20 months.

	males			females		
Strains	Cluster #* mean±SEM (n)	Rel. cluster size mean±SEM (n)	Binary data**	Cluster #* mean±SEM (n)	Rel. cluster size mean±SEM (n)	Binary data**
**129S1/SvImJ**	4.3±0.8 (9)	0.167±0.047 (9)	1	2.2±0.8 (6)	0.035±0.015 (6)	0
**A/J**	nd	nd	nd	2.8±1.5 (6)	nd	nd
**BALB/cByJ** †	2.4±0.3 (8)	0.018±0.003 (8)	0	2.6±1.3 (7)	0.067±0.047 (7)	0
**BTBR T+ ^tf^/J** ‡	12.7±5.0 (3)	0.141±0.079 (3)	0	9.7±1.7 (4)	0.098±0.019 (4)	0
**C3H/HeJ** †	1.0±0.7 (7)	0.022±0.015 (7)	0	4.3±0.8 (6)	0.027±0.010 (6)	0
**C57BL/6J**	4.7±0.7 (10)	0.195±0.061 (10)	1	2.3±0.3 (3)	0.045±0.025 (3)	0
**C57BL/10J**	3.3±1.0 (6)	0.069±0.029 (6)	0	3.5±1.4 (6)	0.027±0.011 (6)	0
**C57BLKS/J** †	0.0±0.0 (7)	0.000±0.000 (7)	0	0.3±0.3 (6)	0.002±0.002 (6)	0
**C57BR/cdJ** §	8.0±1.2 (11)	0.077±0.020 (11)	0	8.3±1.3 (6)	0.201±0.084 (6)	1
**C57L/J** § ‡	18.9±2.2 (13)	0.415±0.055 (13)	1	13.8±2.4 (8)	0.645±0.218 (8)	1
**CBA/J**	2.8±0.5 (4)	0.024±0.006 (4)	0	1.4±0.3 (12)	0.020±0.006 (12)	0
**DBA/2J**	3.8±0.7 (5)	0.068±0.022 (5)	0	5.9±1.6 (7)	0.093±0.023 (7)	0
**FVB/NJ**	3.0±0.9 (4)	0.034±0.025 (4)	0	1.8±0.7 (6)	0.014±0.007 (6)	0
**KK/H1J**	3.3±1.4 (4)	0.044±0.019 (4)	0	2.7±1.1 (6)	0.030±0.013 (6)	0
**LP/J** ‡	8.3±2.1 (9)	0.230±0.066 (9)	1	4.9±0.9 (12)	0.090±0.024 (12)	0
**NON/LtJ** §	6.3±1.9 (8)	0.164±0.073 (8)	1	18.8±1.4 (12)	0.274±0.025 (12)	1
**NZO/H1LtJ**	nd	nd	nd	1.6±0.6 (5)	nd	nd
**NZW/LacJ**	3.3±1.3 (4)	0.035±0.027 (4)	0	4.8±0.6 (8)	0.113±0.038 (8)	0
**P/J** § ‡	9.0±4.0 (4)	0.237±0.079 (4)	1	7.5±1.7 (10)	0.419±0.277 (10)	1
**PL/J**	nd	nd	nd	2.0±0.8 (5)	nd	nd
**RIIIS/J** †	1.8±0.5 (6)	0.008±0.003 (6)	0	0.7±0.3 (10)	0.003±0.001 (10)	0
**SM/J**	3.7±0.9 (7)	0.027±0.010 (7)	0	0.3±0.3 (6)	0.013±0.010 (6)	0
**SWR/J**	5.8±2.9 (5)	0.082±0.047 (5)	0	3.8±1.4 (4)	0.213±0.074 (4)	1

*Cluster #: number of perivascular cell clusters present per renal cross-section.

**“0”: relative cluster size <0.15; “1”: relative cluster size >0.15.

§ strains included in the kinetics analyses: 6, 12 and 20 months.

† strains without TLOs included in lymphatics and liver infiltration analyses.

‡ strains with TLOs included in lymphatics and liver infiltration analyses.

n: number of kidneys/mice analyzed; nd: not determined.

**Figure 2:**
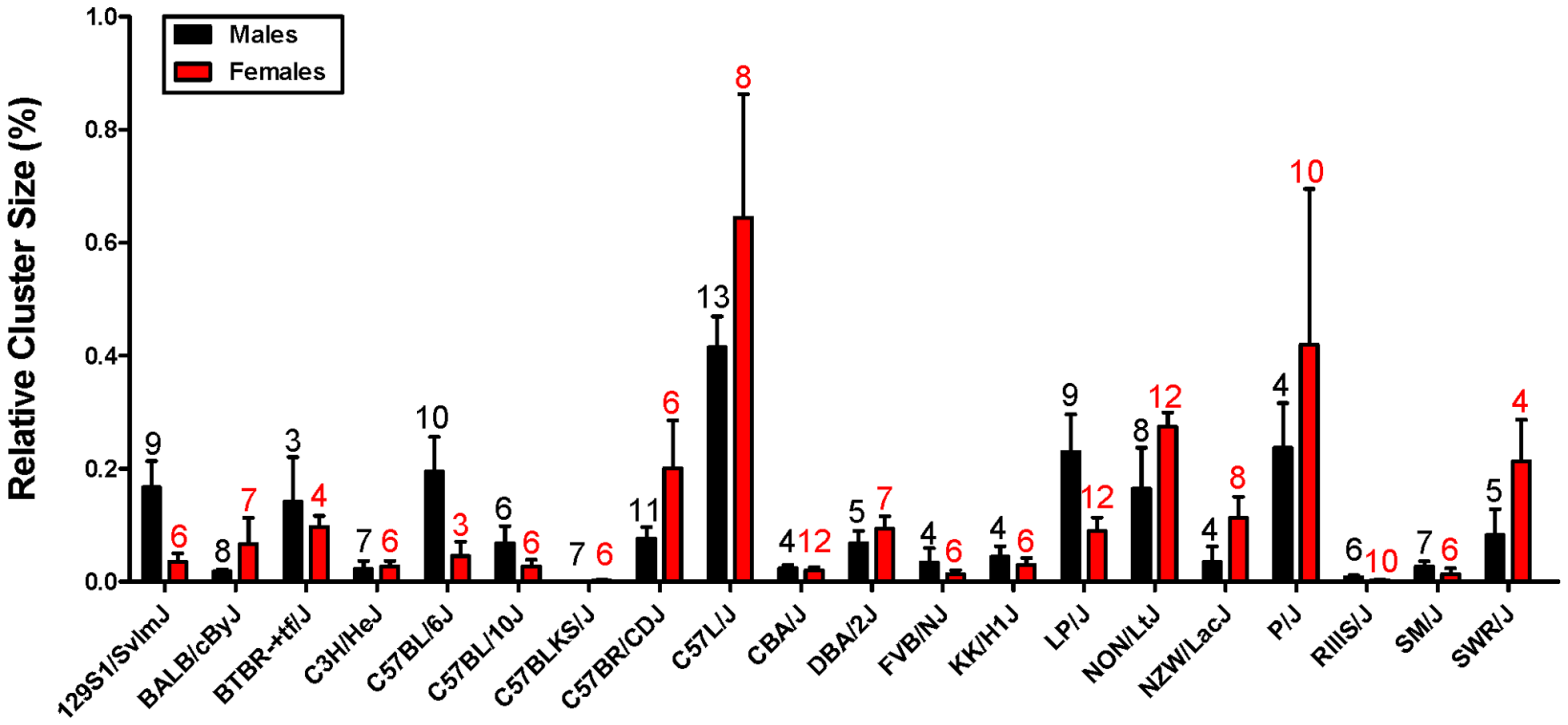
Distribution of relative immune cell cluster size in 20-months-old male and female mice across the different mouse strains. Perivascular immune cell clusters were measured in 20-months-old male and female mice and expressed as relative cluster size (as described in *Materials & Methods*). Only those strains containing both female mice and male mice analyzed were listed (see also Table 1). Data are expressed as mean ± SEM. Numbers indicated above each bar represent the number of mice analyzed.

To analyze the quantitative differences in the distribution of the immune cell clusters among the different strains, the numbers and the size of these clusters were measured. Figure 1A (middle panel) shows a representative example of the surface measurement of three individual immune cell clusters in a PAS-stained section. Table 1 lists the numbers of individual cell clusters as well as the relative cluster size identified in the various strains. To correct for total kidney surface area (which may obviously influence the number of cell clusters present), the relative cluster size in all the strains was calculated using the equation mentioned in the *Materials and Methods* section (Table 1 and Figure 2). These data clearly indicate that the number of individual clusters and the relative cluster size varied among the different strains analyzed. Pearson correlation analyses revealed that the number of clusters identified in both males and females significantly correlated with the relative cluster size (males: Pearson r = 0.8614, P<0.0001 and females: Pearson r = 0.7339, P = 0.0002). As the analysis was stratified by sex, we further looked into the sex difference in all the strains and identified no sex difference with regard to cluster size (P = 0.6716, data not shown). Consequently, both cluster number and the relative cluster size observed in male mice were significantly correlated with those observed in female mice (cluster number: Pearson r = 0.6845, P = 0.0009 & relative cluster size: Pearson r = 0.7972, P<0.0001). Only in strains 129S1/SvlmJ and LP/J, significantly larger clusters were observed in male mice as opposed to female mice. The data were transformed to binary ones (Table 1) with a threshold set at 0.15 in both sexes for HAM analysis (see below).

### Perivascular cell clusters have tertiary lymphoid organ (TLO) characteristics

**Figure 3:**
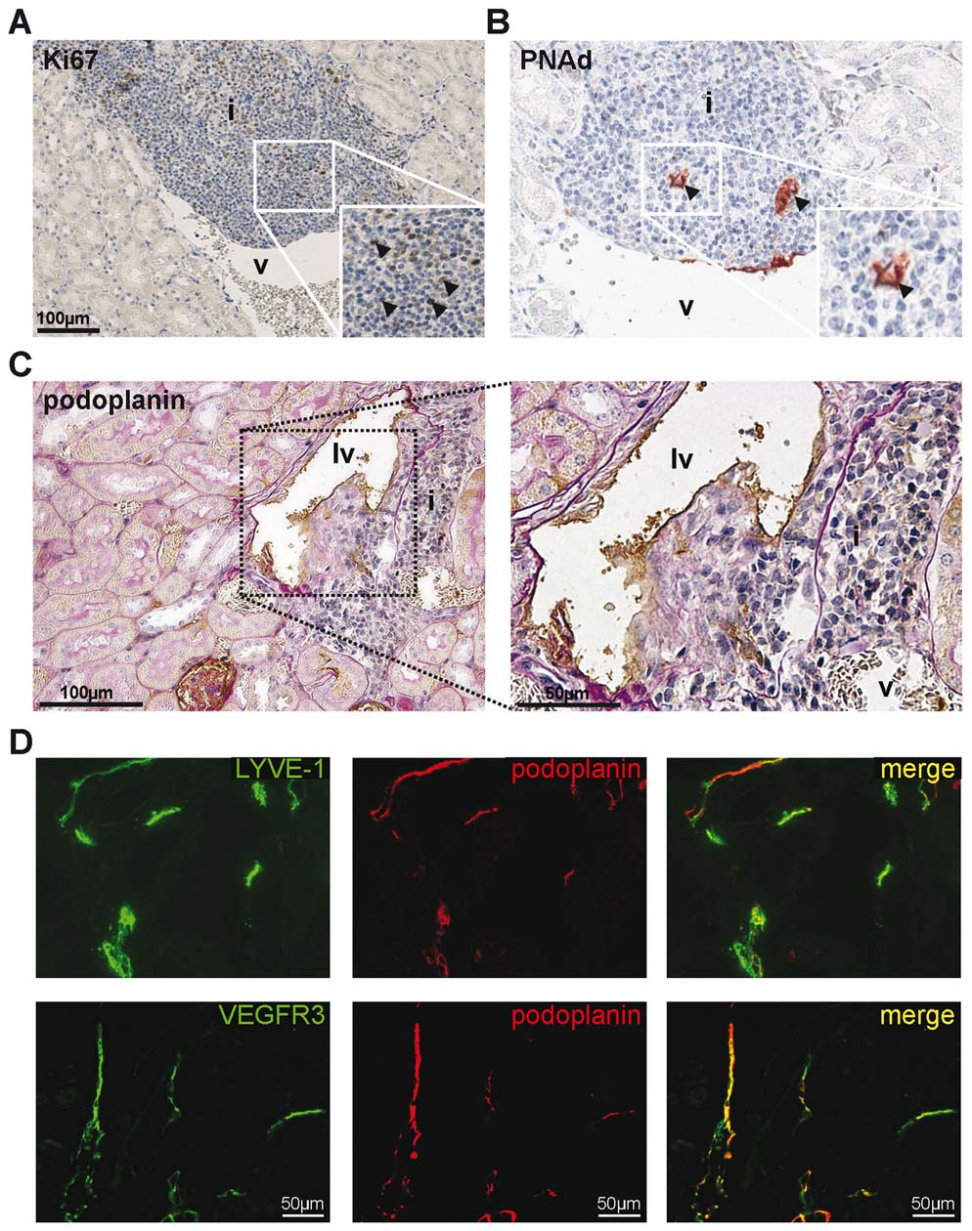
Perivascular immune cell clusters in aged mice have characteristics of tertiary lymphoid organs (TLOs). Representative photomicrographs of a perivascular cell cluster from C57L/J mouse serial sections containing (A) Ki67^+^ proliferating cells (magnification 200x) and (B) peripheral node addressin (PNAd) expressing high endothelial venules (HEVs) (magnification 400×). Insets show higher-power magnifications of the indicated areas. Arrowheads indicate proliferating lymphocytes (A) and PNAd^+^ HEVs (B). (C) Podoplanin expression on lymphatic endothelial cells in a lymph vessel in the close proximity of a perivascular cell cluster (left panel: magnification 200x, right panel: 400x). (D) Immunofluorescent double labeling for LYVE-1/podoplanin (upper row) and VEGFR3/podoplanin (bottom row) on C57Bl/6 mouse kidney sections. Abbreviations: i: infiltrate; lv: lymph vessel; v: vein.

Tertiary lymphoid organ (TLO) formation is associated with the presence of immune cell clusters, which are enriched in mainly T cells and B cells. These conditions recapitulate the cellular conditions required for the formation of TLOs. Therefore we next analyzed the perivascular cell clusters in aged kidneys for the presence of common markers of TLOs. In kidneys from 20 months old mice there was abundant presence of proliferating (Ki67^+^) lymphocytes (Figure 3A) indicating ongoing immune activation in the affected organ [15]. Particularly, high endothelial venules (HEVs) abundantly express peripheral-node addressins (PNAds) which are unique sugar structures on highly glycosylated and sulphated forms of sialomucins. The expression of PNAd in aged mouse kidneys provides more evidence for the formation of TLOs (Figure 3B). Also development of lymph vessels might be associated with the appearance of TLOs [16], [17]. We therefore analyzed whether TLOs in aged mice were associated with podoplanin^+^ lymph vessels within or immediately surrounding TLOs. As shown in Figure 3C, lymph vessels could indeed be observed in the close proximity of TLOs. To confirm the phenotype of lymph vessels, immunofluorescent double labeling was performed for podoplanin and two other lymphatic endothelium markers: LYVE-1 and VEGFR3 (Figure 3D). The presence of proliferating cells, HEVs and lymph vessels collectively indicate that the perivascular lymphoid cell clusters are TLOs.

### Perivascular cell clusters increase during ageing

**Figure 4:**
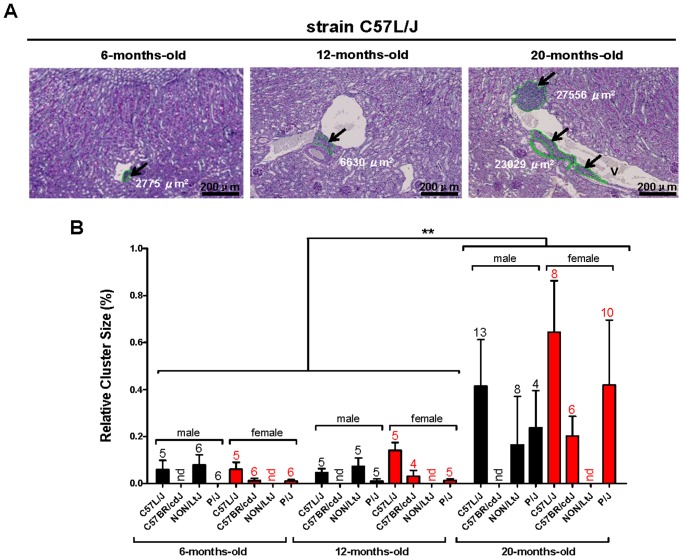
The size of renal perivascular immune cell clusters increase with age. (A) Representative photomicrographs (PAS staining) of perivascular cell clusters in kidneys obtained from C57L/J mice at the age of 6, 12 and 20 months. Cell cluster surface area was calculated and expressed in μm^2^ as indicated (magnification 100x). Arrows indicate individual perivascular infiltrates. Abbreviation: v: vein. (B) Perivascular immune cell clusters were measured in 6-, 12- and 20-months-old male (C57L/J, NON/LtJ, and P/J strains) and female (C57L/J, C57BR/cdJ and P/J strains) mice and expressed as relative cluster size (as described in *Materials & Methods*). Data expressed as mean ± SEM. Numbers indicated above each bar represent the number of mice analyzed. Strains from which no tissues were available are marked as nd (not determined). No differences between 6-months-old and 12-months-old mice in both sexes were observed. However, a significant increase in relative cluster size was observed in 20-months-old mice compared with both 6-months-old and 12-months-old mice (**P<0.01).

To further elucidate the kinetics of perivascular cell cluster and TLO formation during aging, the presence and size of these clusters between aged (20 months) and younger (6 and 12 months) mice were determined in a subset of strains. These strains were selected based on the presence of predominant cell clusters at 20 months and included C57L/J, C57BR/cdJ, P/J and NON/LtJ male and female mice. As shown in Figure 4A and 4B, perivascular cell clusters developed during ageing. Although perivascular cell clusters started to develop already at 6 months of age, no significant differences were observed between 6 and 12 months. At 20 months of age the relative cluster size was significantly increased (P<0.01) compared with 6 and 12 months old mice (Figure 4B).

### TLO formation is characterized by overall reduced numbers of lymphatics

**Figure 5:**
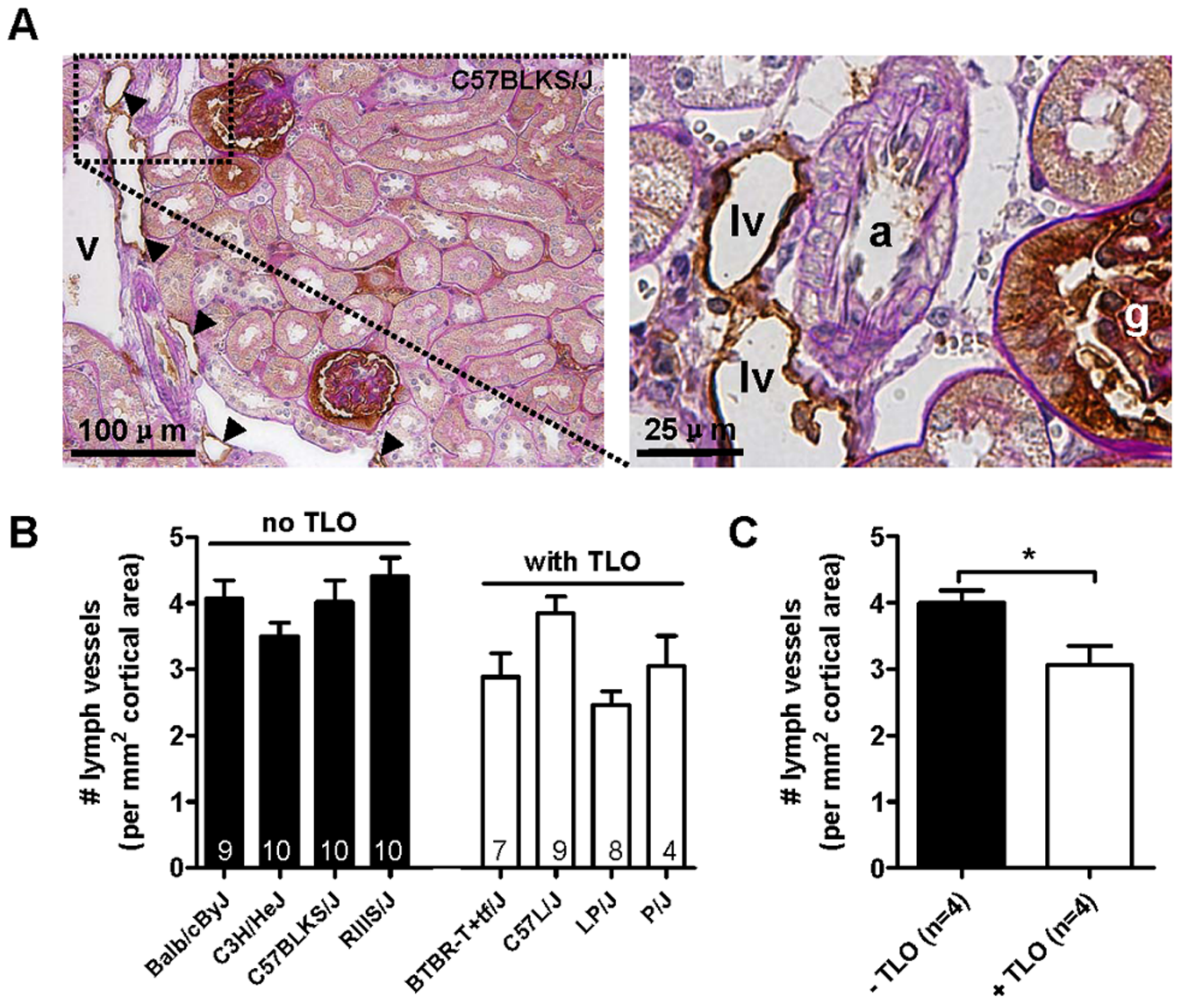
Strains with TLO formation are characterized by reduced numbers of lymph vessels. (A) Representative photomicrograph of peri-arteriolar lymph vessels in a C57BLKS/J mouse kidney (left panel). The right panel shows a high-power magnification of the indicated framed area. Arrowheads indicate podoplanin^+^ lymph vessels. Abbreviations: a: arteriole; g: glomerulus; lv: lymph vessel; v: vein. (B) The number of podoplanin^+^ lymph vessels was quantified in selected strains based on the relative absence (BALB/cByJ, C3H/HeJ, RIIIS/J, C57BLKS/J [black bars]) and abundance (BTBR T+ tf/J, C57L/J, P/J, LP/J [white bars]) of TLOs at the age of 20 months. Numbers indicated within each bar represent the number of mice analyzed. (C) The mean number of lymph vessels in the 4 strains without TLOs (BALB/cByJ, C3H/HeJ, RIIIS/J, C57BLKS/J [black bars]) and with TLOs (BTBR T+ ^tf^/J, C57L/J, P/J, LP/J [white bars]) was calculated. Strains with TLO formation had overall significantly lower numbers of lymph vessels compared with strains without TLOs (*P<0.05).

In order to study whether TLO formation was associated with altered numbers of lymphatics, lymph vessels were stained for podoplanin. Podoplanin has been shown to be one of the most reliable markers for visualization of lymph vessels by immunohistochemistry [18], [19]. To this end, 4 strains with relative absence (BALB/cByJ [n = 9], C3H/HeJ [n = 10], RIIIS/J [n = 10], C57BLKS/J [n = 10]) and 4 strains with relative abundance (BTBR T+ ^tf^/J [n = 7], C57L/J [n = 9], P/J [n = 8], LP/J [n = 4]) of TLOs were analyzed (Table 1). In our study lymph vessels were almost exclusively observed in the adventitia of middle-sized to large arterioles in the cortex and cortico-medullary region of the kidneys analyzed, irrespective of presence or absence of TLOs (Figure 5A). Interstitial lymph vessels were not observed. It is well known that glomerular podocytes and parietal epithelial cells strongly express podoplanin. These components were excluded from the quantitative analysis. In kidneys with TLOs, several lymph vessels were associated with these infiltrates, both around (Figure 2C) and inside TLOs, and some of them filled with lymphocytes (not shown). Although TLO formation appeared to be associated with lymph vessels around or inside TLOs, the overall number of lymph vessels was significantly lower in the kidneys from strains with TLO formation (Figure 5B & 5C).

### Renal TLO formation is associated with perivascular infiltrates in the liver

**Figure 6:**
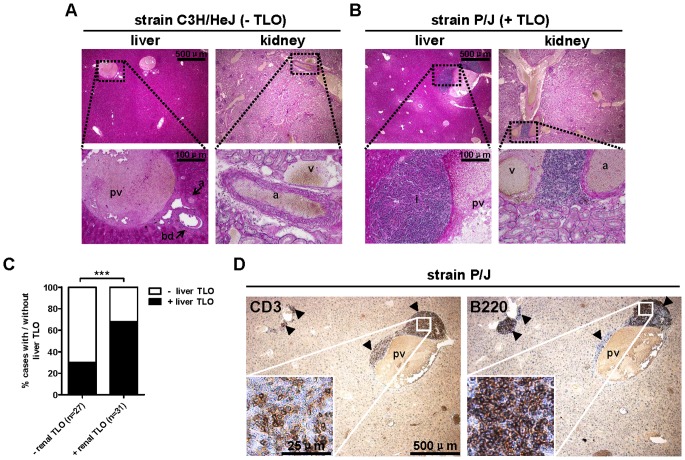
Renal TLO formation is associated with perivascular infiltrates in the liver. Four strains with relative absence (BALB/cByJ [n = 9], C3H/HeJ [n = 10], RIIIS/J [n = 10], C57BLKS/J [n = 10]) and four strains with relative abundance (BTBR T+ ^tf^/J [n = 7], C57L/J [n = 9], P/J [n = 8], LP/J [n = 4]) of renal TLOs were analyzed for the presence of perivascular infiltrates in the liver. (A) PAS staining on C3H/HeJ kidney and liver without perivascular infiltrates (magnifications: 40x and 200x). (B) PAS staining on P/J kidney and liver with perivascular infiltrates (magnifications: 40x and 200x). (C) 21/31 (67.7%) of mice with renal TLOs contained liver TLOs, whereas 8/27 (29.6%) of mice without renal TLOs contained liver TLOs (Pearson's χ^2^ test, P<0.0001). (D) The perivascular infiltrates in liver consisted of CD3^+^ T cells and B220^+^ B cells (magnification: 40x). Insets show high-power magnifications of the indicated areas. Arrowheads indicate positively stained cell clusters. Abbreviations: a: arteriole, bd: bile duct; i: infiltrate; pv: portal vein, v: vein.

We next analyzed whether renal TLO formation is accompanied by inflammatory infiltrates in other organs and analyzed the liver to this end. Again, the 4 strains without (BALB/cByJ, C3H/HeJ, RIIIS/J, C57BLKS/J) and with (BTBR T+ ^tf^/J, C57L/J, P/J, LP/J) TLOs were analyzed (Table 1). In general, in kidneys without TLOs the livers were also devoid of infiltrates (Figure 6A). However, in mice with renal TLO formation, most livers also contained perivascular infiltrates (Figure 6B). Quantitative analysis revealed that 21/31 (67.7%) of mice with renal TLOs also contained liver infiltrates, whereas 8/27 (29.6%) of mice without renal TLOs contained liver infiltrates (Figure 6C). Contingency analysis (Pearson's χ^2^ test) revealed a significant association between renal TLO development and presence of perivascular infiltrates in the liver (χ^2^ = 28.89, P<0.0001). The perivascular infiltrates in liver consisted of predominantly B220^+^ B cells and CD3^+^ T cells (Figure 6D).

### HAM analysis on binary data from male and female mice

**Figure 7:**
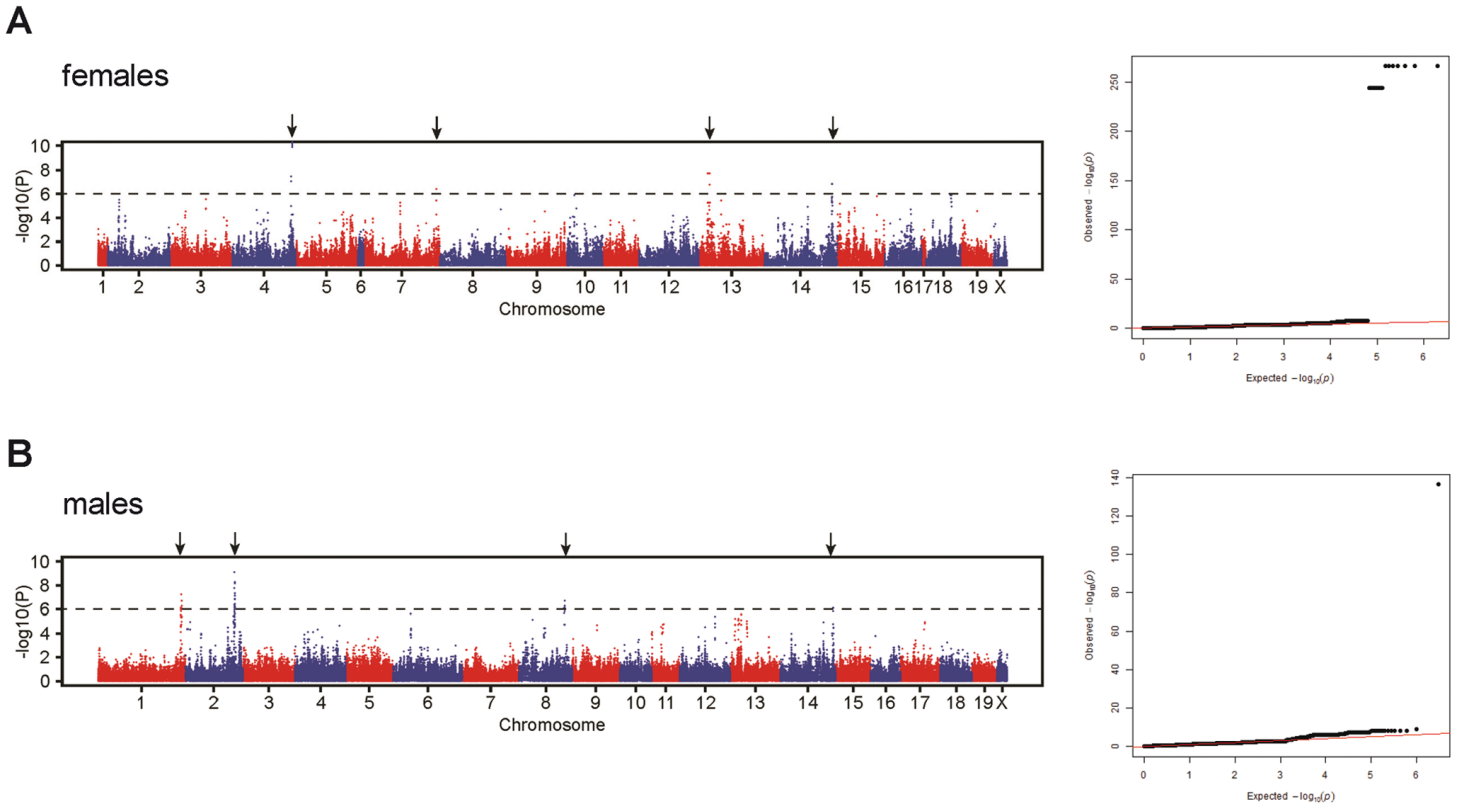
Genome-wide haplotype association mapping in aged mice. In both female (A) and male (B) mice, binary data were used based on the threshold at 0.15 of relative cluster size. Strains with relative cluster size less than 0.15 were marked as “0”, and those higher than 0.15 were marked as “1”. Associations with a P-value of less than 10^−6^ were considered significant. Results are displayed in Manhattan plots (left graphs) and Q-Q plots (right graphs).

In order to identify loci associated with perivascular cell cluster formation, binary data were used for HAM analysis in both sexes (Table 1). Strains with relative cluster size <0.15 were marked as “0”, and those >0.15 were marked as “1”. Genome-wide scanning was performed in all strains at 20 months of age (Figure 7). In male mice, significant associations were found with loci located on Chr1, Chr2, Chr8 and Chr14, while in females associations were found with loci located on Chr4, Chr7, Chr13 and Chr14 (Table 2). Changing the threshold (0.15) for the relative cluster size for any value between 0.1 and 0.2 did not significantly change the associated loci. The HAM analysis data have been submitted to the Mouse Phenotype Database at The Jackson Laboratory (http://phenome.jax.org/) and will be publicly available shortly.

**Table 2:** Summary of the HAM peaks for both sexes at the age of 20 months with a P-value <10^−6^.

Chr	Peak Location*	P-value (M)	P-value (F)	Genes in Interval
1	189,840,009	5.91×10^−9^	0.174	Spata17, Gpatch2, Esrrg, Ush2a
2	163,609,677	5.00×10^−137^	0.900	Ada, Wisp2, Kcnk15, Rims4
8	105,226,793	5.85×10^−8^	0.165	Cdh11
14	118,151,243	5.31×10^−7^	0.62	Gpc6, Abcc4
4	144,880,682	0.566	6.42×10^−267^	Nmnat1, Lzic, Ctnnbip1
4	148,849,737	0.384	5.42×10^−245^	Tnfrsf8
7	118,151,243	0.478	5.31×10^−7^	Asb7, Lins2, Lass3
13	16,065,137	0.082	1.70×10^−8^	Inhba, Cdk13, miR466i, Rala, Pou6f2
14	114,067,017	0.22	1.32×10^−7^	-

Candidate genes are in bold. M: males; F: female.

*NCBIm37 Assembly.

### Correlation of the phenotype with coding SNPs in *Wisp2* and *Tnfrsf8*

The haplotype block containing the gene *Wisp2* showed the strongest association in male mice (P = 5.00×10^−137^), while the haplotype blocks containing *Ctnnbip1* (P = 6.42×10^−267^) and *Tnfrsf8* (P = 5.42×10^−245^) showed the strongest association in female mice. *Wisp2* and *Ctnnbip1* are part of the Wnt-signaling pathway, while *Tnfrsf8* is expressed in activated T and B cells. We therefore analyzed these genes for coding differences among the different inbred strains that would correlate with the phenotype. The Sanger Institute (www.sanger.ac.uk/resources/mouse/genomes/) recently sequenced the complete genomes of 17 inbred strains, which include many of the strains (both with and without clusters) included in our study. The Sanger Institute sequence data show a non-synonymous SNP in exon 4 of *Wisp2* and a non-synonymous SNP in exon 5 of *Tnfrsf8*. No SNPs in the coding regions of *Ctnnbip1* were identified. Subsequently, we determined the genotype for the two SNPs (*i.e*., exon 4 of *Wisp2* and exon 5 of Tnfrsf8) in all the strains included in our survey (Table 3). For the *Wisp2* SNP (rs27315871) which determines the amino acid at position 164 (either R or Q), 64% of the males in strains with a cluster size <0.15 had the R allele, while 100% of strains with a cluster size >0.15 had the Q allele. For the *Tnfrsf8* SNP (rs27627526) which determines the amino acid at position 161 (either G or A), 93% of the females in strains with a cluster size <0.15 had the G allele, while 44% of the strains with a cluster size >0.15 had the A allele.

**Table 3:** *Wisp2* and *Tnfrsf8* alleles identified in the various mouse strains using SNP analysis.

Protein	Allele	Strains
WISP2	R	C57BR/cdJ, C57BL/10J, SWR/J, CBA/J, BALB/cByJ, BTBR *T+ ^tf^*/J,
		C3H/HeJ, FVB/NJ, SM/J
	Q	C57BLKS/J, DBA/2J, KK/J, NZW/LacJ, RIIIS/J, C57L/J, NON/LtJ,
		P/J, LP/J, 129S1/SvImJ, C57BL/6J
TNFRSF8	G	C57BLKS/J, RIIIS/J, FVB/NJ, A/J, C3H/HeJ, C57BL/10J, KK/J,
		129S1/SvImJ, C57BL/6J, BALB/cByJ, DBA/2J, BTBR *T+ ^tf^*/J,
		NZW/LacJ, SWR/J
	A	NZO/H1LtJ, PL/J, CBA/J, SM/J, LP/J, NON/LtJ, P/J, C57BR/cdJ,
		C57L/J

Strains affected are underlined.

### Expression of WISP2, CTNNBIP1 and Tnfrsf8 in perivascular TLOs

**Figure 8:**
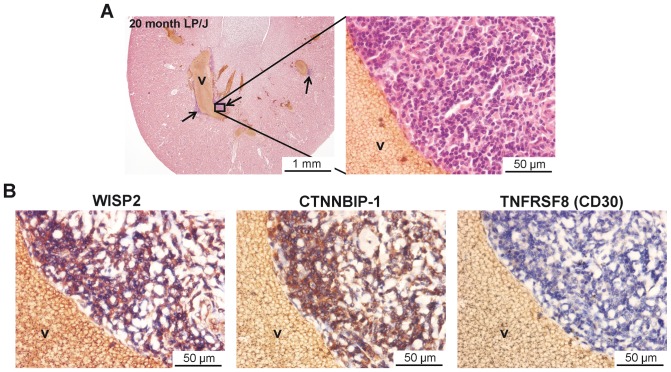
Lymphocytes in renal TLOs express WISP2 and CTNNBIP1, but not TNFRSF8. (A) H&E staining on a kidney section from a 20 month old male LP/J mouse with perivascular infiltrates (TLOs). Magnification: 20x (left panel) and 400x (right panel). (B) Immunohistochemistry revealed expression of WISP2 (left panel) and CTNNBIP1 (middle panel), but not TNFRSF8 (right panel). Magnification: 400x. v: vein.

As described above, we identified various loci in male and female mice that were associated with the development of renal perivascular TLOs of which *Wisp2*, *Tnfrsf8* and *Ctnnbip1* showed the strongest associations. Using immunohistochemistry we identified WISP2 and CTNNBIP1 but not TNFRSF8 protein expression within the perivascular infiltrates (Figure 8).

## Discussion

Immune cell clusters, which developed into TLOs were present in aged kidneys in both male and female mice of a large cohort (≥20) of different inbred mouse strains. These clusters were mainly localized at unique perivascular regions, especially in the area between an arteriole and the accompanying vein. The number and the size of the clusters was closely correlated indicating ongoing recruitment of leukocytes once the process has started. Immune cell infiltration and TLO formation appeared to be a systemic event since we observed a similar process in the liver. The immune cell clusters in aged kidneys exhibited various features of TLOs: aggregation of T and B cells [13], [20], proliferation (Ki67^+^ cells), and presence of PNAd^+^ HEVs [21]. Presence of similar infiltrates in aged mouse kidneys was described before [22]. In that study, some of the kidneys from 3 months old NON mice appeared to have perivascular infiltrates whereas at the age of 12 months all NON mice included had perivascular infiltrates [22].

TLO formation appeared to be negatively correlated with the overall number of lymph vessels. This is remarkable since in renal diseases generally increased numbers of lymph vessels are found [23]. Under physiological conditions, fluid leaves the vascular capillary bed and ends up in the interstitial space after which it enters the lymphatic system together with antigens and leukocytes. Subsequently, the lymph is drained to regional lymph nodes where leukocytes participate in the generation of immune responses. The lymph drainage system becomes especially challenged during disease conditions such as wound healing, inflammation, and infection, when excessive fluid, lymphocytes and dendritic cells travel through the lymphatic system and become activated in response to antigens presented within the lymph nodes. Since most of the lymph vessels were found in the arteriolar adventitia the reduction of the total number of lymph vessels in TLO rich kidneys in our mice might merely be a reflection of the spatial occupation of the TLOs in the adventitia. However, defective lymphatic drainage has also been proposed to be a trigger for lymphoid neogenesis [24], which could indeed imply that strains with relatively lower numbers of lymph vessels are more prone to develop TLOs as observed in our study.

Generally, conditions in which TLO formation can be found include organ-specific autoimmune disorders and other chronic inflammatory and infectious diseases [15] as well as transplant rejection [20], [25], [26]. TLO formation at sites of inflammation or infection is an important part of the local immune response [27] although it is unknown whether TLOs have the same functional properties of secondary lymphoid organs [15]. Irrespective of the potential beneficial effects of TLOs in mounting adequate local immune responses to antigenic stimuli, TLOs are clearly associated with organ specific pathologies [15], [28], [29]. In the kidney, TLO formation is usually associated with chronic rejection [20] and autoimmune diseases [30]. The role of TLO formation during renal aging is unclear but may contribute to aging-related morphological and functional deterioration. However, in our study the presence of TLOs was not correlated with renal function decline (based on microalbuminuria and blood urea nitrogen) when comparing our histological data with historical function data from another cohort [13] of the same strains (not shown). Despite the absence of a correlation between renal function and perivascular TLOs in aged but otherwise healthy mice, increased vulnerability to renal damage in response to a second hit in the presence of TLOs cannot be excluded.

Using HAM analysis, we identified 4 loci in male mice and 5 loci in female mice to be associated with the development of perivascular cell clusters. The loci with *Wisp2*, *Tnfrsf8* and *Ctnnbip1* were the most strongly associated ones. *Wisp2* and *Ctnnbip1* are part of the Wnt-signaling pathway, while *Tnfrsf8* is expressed in activated T and B cells [31] and are therefore considered strong candidate genes. *Wisp2* encodes the protein Wnt-1 inducing signal 2 (WISP2 or CCN5), which is a matricellular protein belonging to the CCN family. WISP2 lacks the cysteine-knot-containing module (which exists in other CCN family members) and contains three functional domains: (i) an insulin-like growth factor binding protein-like module (IGFBP); (ii) a von Willebrand factor type C repeat module (VWC); and (iii) a thrombospondin type-1 repeat module (TSP-1) [32]. WISP2 was previously shown to be associated with the Wnt-1 signaling pathway [33]. Overactivation of Wnt signaling in hepatocellular carcinoma cell lines identified WISP2 as a downstream target of Wnt3A [34]. These data indicate that WISP2 is involved in the Wnt-signaling pathway. In our study, a non-synonymous SNP in *Wisp2* (rs27315871) in exon 4 was found that leads to an amino acid difference among strains (Q164R). Exon 4 encodes the VWC domain of WISP2 which is reported to interact with bone morphogenetic protein [35]. Based on these functions we hypothesize that the Q allele in mice leads to functional differences of WISP2 thereby promoting the development of immune cell clusters and TLOs. However, we identified a few strains (DBA/2J, SWR/J, C57BR/cdJ, and RIIIS/J) that had the Q allele but no perivascular cell clusters. We speculate that other genes in these strains counteract the effect of the Q allele and protect these strains from developing cell clusters and TLOs. Although we were able to demonstrate WISP2 protein expression in perivascular TLOs, further functional studies should focus on the relationship between the functional and the structural differences caused by the Q164R polymorphisms in order to address this issue.

*Ctnnbip1* and *Tnfrsf8* were the two strongest associations detected from the female mice data. Interestingly, *Ctnnbip1* is alike *Wisp2* also involved in the Wnt-signaling pathway. *Ctnnbip1* encodes beta-catenin interacting protein 1 (CTNNBIP1 or ICAT), which is a negative regulator of β-catenin in the Wnt-signaling pathway. CTNNBIP1/ICAT directly inhibits the interaction between β-catenin and TCF4, thus suppressing downstream signaling mediated by β-catenin and TCF4 [36]. Alike WISP2, also CTNNBIP1 was found to be expressed in perivascular TLOs. Although no non-synonymous SNPs in *Ctnnbip1* were identified in our cohort of strains, we cannot exclude differential gene expression that associates with the phenotype. On the other hand, *Tnfrsf8* does contain a non-synonymous SNP (rs27627526). *Tnfrsf8* encodes the protein tumor necrosis factor receptor superfamily member 8 (TNFRSF8 or CD30), which belongs to the tumor necrosis factor receptor (TNFR) superfamily [37]. CD30 is normally expressed in activated T cells and B cells [31] and its expression is upregulated in various hematological malignancies [38]. CD30 is well known for its significant role in the generation of memory T cells, which is involved in the process of maintaining secondary lymphoid tissue structure [39]. Given the common mechanism in the formation of secondary and tertiary lymphoid structures [15], we postulate that CD30 also might have a functional role in TLO formation. This is supported by the observation that CD30 heterozygote *Foxp3^−/−^Ox40^+/−^CD30^+/−^* mice develop an autoimmune phenotype with lymphocytic infiltration and TLO development in the liver whereas CD30 deficient *Foxp3^−/−^Ox40^+/−^CD30^−/−^* mice do not [39]. The SNP (rs27627526) detected in our study determined the amino acid at position 161 (either G or A), which follows the three cysteine-rich motifs in the extracellular domain of the murine CD30 protein [40]. The functional role of this amino acid difference is still unknown. Despite our efforts, no TNFRS8 expression was detected within the TLOs.

It is well known that in experimental models both genetic background and sex play an important role in the process of aging-related renal morphological and functional deterioration [41]. We also observed strong strain differences regarding the presence of perivascular immune cell clusters and TLOs. These differences could be partly explained by the genes and SNPs detected, and underscore the significant role of genetic background in these processes. When comparing the renal phenotypes across the different inbred strains, we separately analyzed female and male mice to detect potential gender-related differences. Although relative cluster size and cluster number between female and male mice were correlated, not all strains displayed this correlation in particular when analyzing the binary data. Based on the binary data, in total 8 strains were identified with TLOs of which only 3 had TLOs in both the male and female mice. Based on these differences between males and females, we performed HAM analyses on males and females separately. Although these analyses revealed different loci, the candidate genes detected in both sexes are involved in the Wnt-signaling pathway.

In conclusion, we found the presence of perivascular immune cell clusters and TLOs in aged mouse kidneys. The clusters develop in time and relative cluster size differs among the various strains being relatively consistent between males and females. Among the genes detected by HAM analysis, *Wisp2* (male mice) and *Ctnnbip1* and *Tnfrsf8* (female mice) are strong candidate genes based on their reported functions. These candidates are involved in the Wnt-signaling pathway and may be causally linked to aging-related inflammation and TLO formation.
